# De Novo Donor-Specific HLA Antibodies Developing Early or Late after Transplant Are Associated with the Same Risk of Graft Damage and Loss in Nonsensitized Kidney Recipients

**DOI:** 10.1155/2017/1747030

**Published:** 2017-03-06

**Authors:** Michela Cioni, Arcangelo Nocera, Annalisa Innocente, Augusto Tagliamacco, Antonella Trivelli, Sabrina Basso, Giuseppe Quartuccio, Iris Fontana, Alberto Magnasco, Francesca Drago, Antonella Gurrado, Ilaria Guido, Francesca Compagno, Giacomo Garibotto, Catherine Klersy, Enrico Verrina, Gian Marco Ghiggeri, Massimo Cardillo, Patrizia Comoli, Fabrizio Ginevri

**Affiliations:** ^1^Nephrology, Dialysis and Transplantation Unit, IRCCS Istituto G. Gaslini, Genova, Italy; ^2^Clinical Nephrology Unit and Transplant Immunology Research Laboratory, Department of Internal Medicine, University of Genova and IRCCS San Martino University Hospital IST, Genova, Italy; ^3^Transplantation Immunology, IRCCS Fondazione Ca' Granda, Ospedale Maggiore Policlinico, Milano, Italy; ^4^Pediatric Hematology/Oncology and Cell Factory, Fondazione IRCCS Policlinico S. Matteo, Pavia, Italy; ^5^Vascular and Endovascular Unit and Kidney Transplant Surgery Unit, University of Genova, IRCCS San Martino University Hospital IST, Genova, Italy; ^6^Biometry and Statistics Service, Fondazione IRCCS Policlinico S. Matteo, Pavia, Italy

## Abstract

De novo posttransplant donor-specific HLA-antibody (*dn*DSA) detection is now recognized as a tool to identify patients at risk for antibody-mediated rejection (AMR) and graft loss. It is still unclear whether the time interval from transplant to DSA occurrence influences graft damage. Utilizing sera collected longitudinally, we evaluated 114 consecutive primary pediatric kidney recipients grafted between 2002 and 2013 for* dn*DSA occurrence by Luminex platform.* dn*DSAs occurred in 39 patients at a median time of 24.6 months. In 15 patients,* dn*DSAs developed within 1 year (*early-onset* group), while the other 24 seroconverted after the first posttransplant year (*late-onset* group). The two groups were comparable when considering patient- and transplant-related factors, as well as DSA biological properties, including C1q and C3d complement-binding ability. Only recipient age at transplant significantly differed in the two cohorts, with younger patients showing earlier* dn*DSA development. Late AMR was diagnosed in 47% of the* early* group and in 58% of the* late *group. Graft loss occurred in 3/15 (20%) and 4/24 (17%) patients in* early-* and* late-onset* groups, respectively (*p* = ns). In our pediatric kidney recipients,* dn*DSAs predict AMR and graft loss irrespective of the time elapsed between transplantation and antibody occurrence.

## 1. Introduction

Humoral alloimmunity leading to chronic antibody-mediated rejection (AMR) has been recognized as a major obstacle to long-term kidney graft (KTx) survival [1–5]. In addition to sensitized patients who suffer from poorer kidney graft outcome due to a higher incidence of AMR, a number of pretransplant HLA-antibody-negative kidney recipients, usually considered at low immunological risk, will also develop chronic allograft dysfunction and, ultimately, graft loss [6–16]. A positive association between the presence of de novo posttransplant donor-specific HLA antibodies (*dn*DSAs) after transplantation and poor transplant outcome has been demonstrated also in this patient category. This observation has prompted recommendations on posttransplant HLA-antibody monitoring as a tool to identify patients at risk for antibody-mediated rejection and graft loss [6–16].

It has also been shown that DSA development continues as an active process even many years after transplantation [12, 14–17], and although DSAs may be detected also in patients with long-term functioning allografts, persistent kidney loss due to antibody-mediated injury is observed throughout the whole posttransplant period [5]. Time to DSA development has been suggested as a variable that could impact transplant outcome, with* early*-onset DSAs being associated with lower graft survival [7], but data on this clinical scenario are not conclusive [9, 17].

We conducted a longitudinal analysis on a pediatric cohort of pretransplant HLA-antibody-negative, first kidney recipients sequentially monitored for posttransplant DSA onset and alloantibody biological properties, in order to evaluate whether the timing of* dn*DSA appearance could influence AMR development and graft outcome.

## 2. Patients and Methods

### 2.1. Patients

Between July 2002 and March 2013, 125 consecutive patients were referred to the Genoa Pediatric Kidney Transplant Program for first allografting. Pretransplant patient sera were screened periodically for the presence of panel reactive anti-HLA antibodies by complement dependent cytotoxicity technique and by a bead-based assay [12]. All grafts were performed after a negative T cell crossmatch. Our standard of care for low immunological risk kidney transplant patients consisted of induction with basiliximab and a triple drug immunosuppressive regimen including a calcineurin inhibitor (cyclosporin A or tacrolimus), mycophenolate mofetil, and prednisone. Biopsy-proven acute cellular rejection episodes were treated with pulse intravenous methylprednisolone. Patients developing late AMR, as evidenced by circulating HLA DSAs and histological features of antibody-mediated tissue and vascular injuries, were treated with a protocol including a combination of plasmaphereses, i.v. human Ig, and anti-CD20 monoclonal antibody. Graft function was estimated by calculating eGFR using the Schwartz [18] or MDRD [19] formula, when appropriate.

Graft biopsies were performed for clinical indication (graft function decline and/or proteinuria); since 2010, DSA positivity was also included among indications. Rejections were histologically graded following the Banff 97 criteria with updates. Banff 2009 and Banff 2013 criteria were employed for classifying C4d positive and negative AMR [20, 21]. All biopsies performed before 2014 were regraded according to the Banff 2013 criteria. C4d staining was performed on frozen sections by indirect immunofluorescence.

This study was approved by the Institutional Review Board of the Fondazione Ca' Granda, Ospedale Maggiore Policlinico, Milano (867/2014).

### 2.2. Detection and Characterization of HLA Antibodies

Recipients of first graft who were found positive for the presence of anti-HLA antibodies in current and/or historical pretransplant sera (*n* = 11) were not included, resulting in a total of 114 nonsensitized first kidney allograft pediatric recipients monitored for* dn*DSA (Table 1). Sera for HLA-antibody monitoring were collected at transplantation, every three months in the first posttransplant year and annually thereafter. Samples obtained before 01/2010 belonged to a unique source of sera analyzed retrospectively for HLA antibodies, while from 02/2010 all samples were collected and analyzed prospectively [12]. An average of >8 samples per patient were analyzed. Complement-binding activity was analyzed on sera collected at DSA appearance and at biopsy or at follow-up.

HLA typing of kidney graft recipients and donors was performed as previously described [22]. Anti-HLA class I and class II IgG antibodies were tested with a bead-based detection assay after serum EDTA treatment, to avoid underestimation of antibody MFI strength [23–25]. We used the LABScreen Mixed kit and the Single-Antigen Bead (SAB) assays (One Lambda Inc., CA, USA) to identify HLA class I and class II specificities [12, 22]. Screening assay results above a cut-off value of 3.0 ratio between the sample and negative control were considered positive. Single-antigen results above a MFI cut-off value of 1.000 were considered positive. Heat inactivated patient sera were tested with C1qScreen™ (One Lambda) for identification of complement-binding antibodies, as described [26]. Antibody positivity was assigned at >500 MFI. Serum samples were analyzed in a blinded fashion for the presence of C3d-binding DSA with the single-antigen flow bead technology, according to the manufacturer's protocol (Immucor Lifecode Transplant Diagnostics Nijlen, Belgium). Positivity was assigned as previously detailed [27].

### 2.3. Statistical Analysis

Data were described as the mean and standard deviation (SD) or median and range if continuous and as count and percent if categorical. To determine differences among patient groups, categorical variables were compared by chi-squared analysis, continuous variables with *t*-tests, and, if skewed, nonparametric tests (Kruskal-Wallis one-way analysis of variance, Mann–Whitney *U* test). *p* values < 0.05 were considered statistically significant. Event-free survival was estimated with the Kaplan-Meier method and was compared between risk groups with the log-rank test. For graft failure, censoring event was death with functioning graft. For AMR, censoring event was graft failure. Patients who did not experience graft failure or AMR were censored at the end of the follow-up. Stata 13 (Stata Corporation, College Station, TX, USA) or the NCSS System (NCSS, Cary, NC) was used for computation.

## 3. Results

### 3.1. Clinical and Immunological Characteristics of the Patients according to Time of* dn*DSA Development

The cohort median follow-up was 6.7 years (range 2.0–12.6). Antibody identification was based on longitudinal analysis of collected sera in both retrospective and prospective sample series. Among the 114 patients analyzed, 39 patients (34%) developed* dn*DSAs at a median time of 24.6 months (range 3–115 months). The mean number of DSA specificities found per patient was 1.97 (±1.29).

*dn*DSA-positive KTx recipients were stratified in two groups, based on time to DSA appearance. We considered patients with antibody occurrence within the first 12 months (the period of greater immunosuppression reduction) as those more prone to mount an immune response to the graft (*early-onset* group, *n* = 15) and patients with antibody occurrence beyond the first posttransplant year as the* late-onset* group (*n* = 24) (Table 1). The median time of DSA appearance from transplantation was 9 months (range 3–12) in the early group and 47 months (range 17–115) in the late group. The two groups were comparable when considering patient- and transplant-related factors, such as recipient sex, living versus deceased donor graft source, cyclosporine or tacrolimus administration, delayed graft function, 1-year estimated glomerular filtration rate (eGFR), HLA class I and II mismatches, and incidence of T cell mediated rejection (TCMR) and late AMR. Only recipient age at transplant was found to be significantly different in the two cohorts, with younger patients showing earlier* dn*DSA development (Table 1).

**Figure 1:**
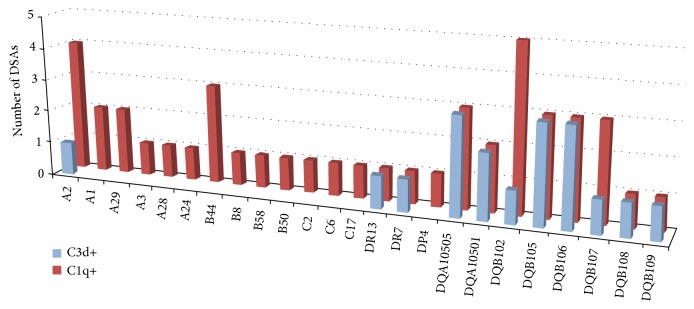
HLA antigens recognized by C3d and/or C1q positive DSAs in the 39* dn*DSA-positive patients. A total of 78* dn*DSAs were identified in the 39 kidney recipients. Of those, 44 bound C1q and 18 displayed binding ability for C3d.

Patients belonging to the two groups did not display any difference in all analyzed HLA-antibody characteristics, including HLA class and locus specificity, persistence, MFI, and C1q and C3d complement fraction binding ability (Table 2). Antibodies detected in* dn*DSA-positive patients recognized a total of 78 HLA antigen specificities. In the two patient groups, HLA class I and class II specificities were equally distributed, and a similar pattern was observed when the analysis was carried out for each HLA antigen locus (Table 3). As observed in the whole cohort, DQ* dn*DSAs were the most represented antibodies in both groups. Regarding DSA biological properties, such as MFI and C1q- and C3d-binding ability, no significant differences were observed in the two groups (Table 3). However,* dn*DSAs differed for their complement-binding capability, as, with the exception of HLA A2, all C3d-positive DSAs recognized HLA class II and, in particular, DQ antigens, while C1q-positive DSAs were homogeneously distributed between the two classes (Figure 1). All C3d-binding DSAs were also found to bind C1q.

### 3.2. Time to* dn*DSA Emergence and Correlation with AMR and Clinical Outcome

**Figure 2:**
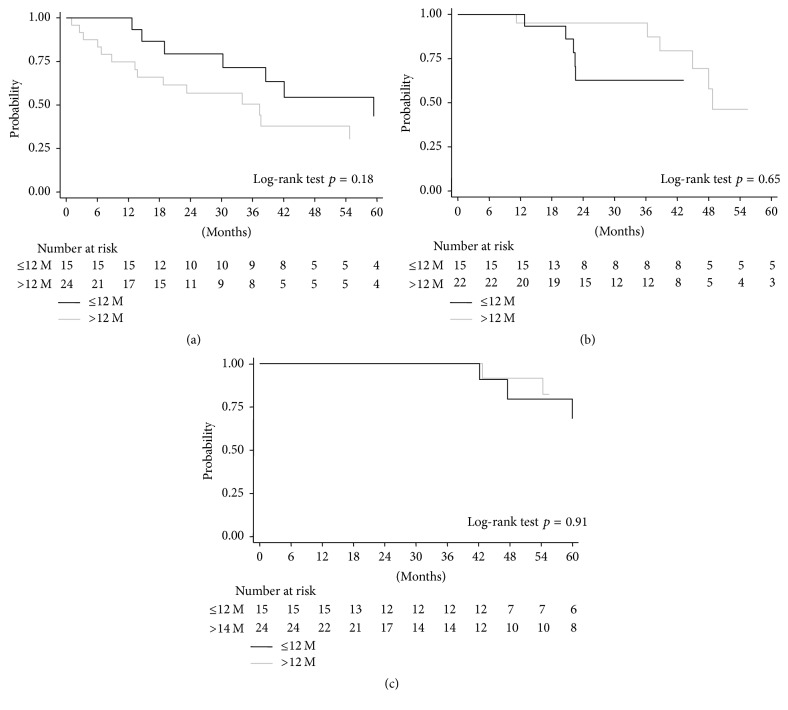
Risk of developing late antibody-mediated rejection (AMR), renal function decline, and graft loss, in the 39 patients who developed de novo donor-specific antibodies (*dn*DSAs), according to the time to HLA-antibody occurrence. (a) AMR-free allograft survival in kidney graft recipients, stratified by early or late development of* dn*DSAs; (b) renal graft function decline (eGFR ≤ 50 ml/min/1.73 m^2^) in kidney graft recipients, stratified by early or late development of* dn*DSAs; (c) allograft survival in kidney graft recipients, stratified by early or late development of* dn*DSAs. The statistical difference between Kaplan-Meier survival curves was evaluated by the log-rank test and differences with *p* values < 0.05 were considered statistically significant.

AMR was diagnosed in 21 patients at a median follow-up of 4.8 years from kidney transplantation and was observed only in patients positive for* dn*DSAs (Table 1). Considering BANFF 2013 criteria for classification of AMR, 10 were acute active, and 11 were chronic active. The distribution of acute active and chronic active AMR did not differ between the* early-* and* late-onset groups*. To evaluate the damaging effect of* dn*DSAs on the kidney graft, we analyzed the rate of AMR-free survival from the time of DSA onset. The interval from* dn*DSA development to AMR was 2.5 years (range 1.0–4.9) in the* early-onset *group, compared to 1.1 years (range 0.1–4.6; *p* = 0.08) in the* late-onset* group. AMR-free survival did not differ between* early-* and* late-onset groups* (Figure 2(a)).

**Figure 3:**
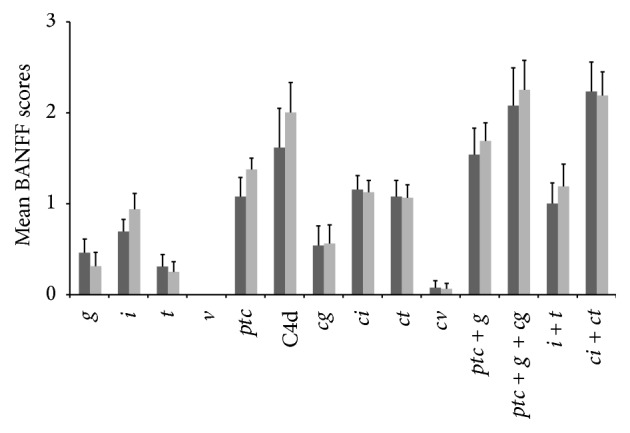
Histological analysis in 30 graft biopsies obtained from 13 recipients displaying* early-onset dn*DSAs (dark grey bars) and 17 recipients positive for* late-onset dn*DSAs (light grey bars). The biopsy findings were analyzed both individually (*i, t, ptc, g, ci, ct, cg, cv, v*) and in functional clusters (*ptc + g* referring to microcirculation inflammation,* ptc + g + cg* to microcirculation lesions,* i + t* to tubulointerstitial inflammation, and* ci + ct* to tubulointerstitial scarring). Data are presented as the mean ± standard error. For each parameter, no significant difference was observed between the two groups.

The histological findings were investigated in graft biopsies obtained from 30 out of 35 patients with persistent* dn*DSAs (Figure 3); for the remaining 5 graft recipients, no biopsies were available, as the patients refused the procedure due to stable good allograft function. The histological findings were analyzed both individually (interstitial inflammation-*i*-, tubulitis-*t*-*, ptc, *glomerulitis-*g*-, interstitial fibrosis-*c*-, tubular atrophy-*ct*-, transplant glomerulopathy-*cg*-, chronic vascular changes-*cv*-, and intimal arteritis-*v*-) and in functional clusters (*ptc + g* referring to microcirculation inflammation,* ptc + g + cg* to microcirculation lesions,* i + t* to tubulointerstitial inflammation, and* ci + ct* to tubulointerstitial scarring). No significant differences were observed between the two groups (Figure 3).

We then evaluated the impact of* early-* versus* late-onset dn*DSAs on graft loss. In the whole cohort of 114 patients, 9 grafts were lost, among which 7 grafts were lost due to AMR and 2 to focal glomerulosclerosis recurrence. The latter 2 patients were* dn*DSA negative. Among the 7 graft losses due to AMR, 3 were observed in the* early-onset* group and 4 in the* late-onset dn*DSA group. The median time interval from* dn*DSA onset to graft loss was 4.0 years (range 3.5–5.0) in the* early-onset *group, compared to 5.5 years (range 3.6–6.5) in the* late-onset *group (*p* = ns) (Figure 2(c)). As the number of graft losses in our cohort was limited, eGFR ≤ 50 ml/min/1.73 m^2^ was alternatively employed as an outcome end-point. Also in this case, no difference was observed between the* early-onset* and* late-onset* groups (Figure 2(b)).

## 4. Discussion

The problem of clarifying whether HLA antibodies developing at different posttransplant intervals could have different cytotoxic capabilities and graft tissue damage potential has relevance in view of the need to establish the optimal terms of posttransplant DSA surveillance strategy, particularly concerning monitoring length.

Our study, carried out in a homogeneous patient population not including sensitized recipients, demonstrates that the time interval to AMR development and graft loss, evaluated from the first* dn*DSA appearance, does not differ in the* early- *and* late-onset* HLA-antibody groups. In previous studies, it had been shown that DSAs developing within the first year after transplantation resulted in early graft failure, whereas* late-onset *DSAs, although also detrimental, seemed to require a longer time to finally cause graft damage and loss [7, 9]. These latter observations likely reflected the presence of a proportion of sensitized patients, in whom rapid development of DSA-mediated tissue damage could have been sustained by the presence of a cytokine inflammatory milieu [28, 29] and further amplified by a parallel action of non-DSAs specific for mismatched cross-reactive epitopes [8]. In our cohort of pediatric recipients, a model intrinsically free of relevant comorbidities, a thorough and prolonged posttransplant antibody monitoring permitted an accurate estimate of the interval between DSA onset and graft function deterioration, thus allowing assessment of the actual damaging potential of* dn*DSAs emerging in the late posttransplant period. Through this longitudinal detection approach, we demonstrated that DSAs in the two patient groups displayed equivalent damaging capabilities. Indeed,* early- *and* late-onset dn*DSAs did not differ in the biological properties, such as high MFI values and complement-binding ability, recently demonstrated to be the main determinants of antibody-mediated graft damage and loss [26, 27, 30–32]. In particular, all of the graft losses in both groups were observed in patients displaying DSAs capable of C3d binding, as a result of progressive acquisition over time of C1q- and C3d-fixing ability, paralleled by an increase in MFI values [27]. The size of our pediatric cohort, smaller than average adult series, may have partly influenced our statistical findings and limited our ability to dissect the respective role of complement-binding activity and MFI on graft outcome. While Lee et al. observed an earlier production of HLA class I DSAs [7], we found that HLA class I and class II* dn*DSAs were comparably represented in both* early- *and* late-onset* groups. This apparent discrepancy could be in part explained by the fact that our study exclusively analyzed nonsensitized recipients. Indeed, in a first set alloresponse condition, the ubiquitous cellular expression of class I HLA antigens within the kidney graft tissue may be balanced by the greater stimulating capability of the highly polymorphic class II molecules, in particular HLA DQ antigens [11–15, 22]. Moreover, comparing C1q- and C3d-binding capabilities in class I and class II* dn*DSAs, we demonstrated in both patient groups that C3d binding was almost exclusively a property of class II, whereas C1q binding was expressed in a similar percentage by both classes. This finding gives additional strength to previous data demonstrating that, in nonsensitized low-risk kidney recipients, class II specific and, in particular, anti-DQ de novo antibodies are the principal effectors of graft loss in all posttransplant phases [11–15, 22, 27, 30]. The equivalency of* early *and* late dn*DSA damaging capacity was further supported by the observation that the two study groups displayed a similar histological pattern of tissue graft damage. In this regard, it is worth underlining that, in our cohort represented by recipients of grafts from young donors, the susceptibility to HLA-antibody mediated insult is only marginally influenced by organ ageing [33].

At present, the reasons for* dn*DSA production in some patients and not in others, as well as the biological factors influencing their development at different times after transplant, in the presence of the same degree of HLA mismatching and the same immunosuppressive regimen, are not completely understood. In this study, younger recipient age appeared to favor an earlier* dn*DSA production, likely reflecting a propensity to display a stronger alloreactivity, that suggests a note of caution in immunosuppressive therapy minimization in the pediatric setting.

## 5. Conclusions

Based on our findings, management of patients found positive for* dn*DSAs at late phases of posttransplant follow-up should not differ from that applied in the early-onset* dn*DSA patient group.

Thus, monitoring of HLA antibodies throughout the entire posttransplant course is recommended, despite high costs and organization difficulties, in order to identify patients at risk for AMR and graft loss.

## Figures and Tables

**Table 1:** Clinical features of the patients analysed and according to the date of de novo donor specific HLA antibody (*dn*DSA) onset.

Variables	All patients (*n* = 114)	All patients with *dn*DSAs (*n* = 39)	Patients with *dn*DSAs occurring within year 1 (*n* = 15)	Patients with *dn*DSAs occurring beyond year 1 (*n* = 24)	*p* value
Characteristics at Tx					
Recipient					
Male sex	69 (60.5%)	27 (69%)	12 (80%)	15 (62.5%)	0.30
Age	13.4	13.20	10.27	15.03	**<0.05**
Donor					
Male sex	70 (61.4%)	25 (64%)	11 (73%)	14 (58%)	0.50
Age	17.2	13.15	11.53	14.17	0.46
Deceased	97 (85%)	36 (92%)	14 (93%)	22 (92%)	1
Transplantation					
Number of total HLA A, B mismatches	2.36	2.56	2.47	2.63	0.70
Number of total HLA DR, DQ mismatches	1.61	1.85	2.00	1.75	0.42
Number of total HLA A, B, DR, DQ mismatches	3.97	4.41	4.47	4.38	0.81
Characteristics after Tx					
CyA in maintenance IS	66 (58%)	30 (77%)	10 (67%)	20 (83%)	0.27
Delayed graft function	13 (11%)	4 (10%)	1 (7%)	3 (12%)	0.50
Acute T cell-mediated rejection^*∗*^	18 (16%)	8 (20%)	2 (13%)	6 (25%)	0.45
eGFR < 60 at 1 year	14 (12%)	7 (18%)	1 (7%)	6 (25%)	0.21
AMR	21 (18%)	21 (54%)	7 (47%)	14 (58%)	0.52

^*∗*^Including borderline changes.

Tx: transplantation; CyA: cyclosporin A; IS: maintenance immunosuppression; eGFR: estimated glomerular filtration rate (ml/min/1.73 m^2^).

**Table 2:** Antibody characteristics in 39 de novo donor specific HLA antibody (*dn*DSA) positive patients.

Variables	All patients with *dn*DSAs (*n* = 39)	Patients with *dn*DSAs occurring within year 1 (*n* = 15)	Patients with *dn*DSAs occurring beyond year 1 (*n* = 24)	*p* value
*dn*DSA specificities, nr/patient^*∗*^	1.97 ± 1.29	1.87 ± 1.25	2.04 ± 1.33	0.68
Persistent^*∗∗*^*dn*DSAs	35 (90%)	13 (87%)	22 (92%)	0.63
HLA class I *dn*DSAs	8 (21%)	2 (13%)	6 (25%)	0.45
HLA class II *dn*DSAs	18 (46%)	8 (53%)	10 (42%)	0.52
HLA class I and II *dn*DSAs	13 (33%)	5 (33%)	8 (33%)	1.00
HLA-A *dn*DSAs	16 (41%)	5 (33%)	11 (61%)	0.51
HLA-B *dn*DSAs	12 (31%)	3 (20%)	9 (37%)	0.30
HLA-C *dn*DSAs	7 (18%)	3 (20%)	4 (17%)	1.00
HLA-DR *dn*DSAs	6 (15%)	3 (20%)	3 (12%)	0.66
HLA-DQ *dn*DSAs	28 (72%)	11 (73%)	17 (71%)	1.00
HLA-DP *dn*DSAs	1 (3%)	0	1 (4%)	1.00
Immunodominant *dn*DSAs				
MFI at onset^*∗*^	9501 ± 7198	10483 ± 7020	8888 ± 7387	0.51
MFI at biopsy or peak^*∗*^	12043 ± 7842	12061 ± 6683	12031 ± 8626	0.99
C1q positivityof* dn*DSAs				
At *dn*DSA onset	25 (64%)	12 (80%)	13 (54%)	0.17
At biopsy or MFI peak	29 (74%)	12 (80%)	17 (71%)	0.71
C3d positivityof* dn*DSAs				
At *dn*DSA onset	9 (23%)	3 (20%)	6 (25%)	1.00
At biopsy or MFI peak	16 (41%)	6 (40%)	10 (42%)	1.00

Percentages are calculated on the total number of patients from each group indicated at the top of the respective columns.

All data are reported as absolute numbers, unless otherwise specified; ^*∗*^data reported as mean ± sd.

^*∗∗*^DSA persistence was defined as positivity of the immunodominant DSA in all analyzed samples after first positivity.

MFI: mean fluorescence intensity.

**Table 3:** Characteristics of 78 de novo donor specific HLA antibodies (*dn*DSAs) detected in 39 DSA positive kidney recipients.

Variables	Total number of *dn*DSAs (*n* = 78)	*dn*DSAs occurring within year 1 (*n* = 26)	*dn*DSAs occurring beyond year 1 (*n* = 52)	*p* value
HLA class I *dn*DSAs	40 (51%)	11 (42%)	29 (56%)	0.34
HLA class II *dn*DSAs	38 (49%)	15 (58%)	23 (44%)	0.34
HLA class I *dn*DSAs, MFI^*∗*^	4678 ± 4516	4838 ± 4717	4618 ±4521	0.89
HLA class II *dn*DSAs, MFI^*∗*^	12033 ± 8410	10629 ± 7568	12949 ±8960	0.41
HLA-A *dn*DSAs^*∗∗*^	18 (23%)	5 (19%)	13 (25%)	0.78
HLA-B *dn*DSAs^*∗∗*^	15 (19%)	3 (11%)	12 (23%)	0.36
HLA-C *dn*DSAs	7 (9%)	3 (11%)	4 (7%)	0.68
HLA-DR *dn*DSAs^*∗∗*^	7 (9%)	4 (15%)	3 (6%)	0.21
HLA-DQ *dn*DSAs^*∗∗*^	30 (38%)	11 (42%)	19 (36%)	0.63
HLA-DP *dn*DSAs	1 (1%)	0	1 (2%)	1.00
C1q binding of* dn*DSAs	44 (56%)	17 (65%)	27 (52%)	0.33
HLA class I *dn*DSAs	20 (26%)	7 (27%)	13 (25%)	1.00
HLA class II *dn*DSAs	24 (31%)	10 (38%)	14 (27%)	0.31
C3d binding of* dn*DSAs	18 (23%)	7 (27%)	11 (21%)	0.58
HLA class I *dn*DSAs	1 (1%)	1 (4%)	0	0.33
HLA class II *dn*DSAs	17 (22%)	6 (23%)	11 (21%)	1.00

Percentages are calculated on the total number of antibodies from each group indicated at the top of the respective columns. All data are reported as absolute numbers, unless otherwise specified.

^*∗*^Data reported as mean ± sd.

^*∗∗*^The number of antibodies detailed in this table is higher than that reported in Table 2, as some patients have multiple DSAs at this locus.

MFI: mean fluorescence intensity.
